# Is cell regeneration and infiltration a double edged sword for porcine aortic valve deterioration? A large cohort of histopathological analysis

**DOI:** 10.1186/s12872-022-02776-6

**Published:** 2022-07-28

**Authors:** Li Li, Xuejing Duan, Hongyue Wang, Yang Sun, Wei Zhao, Yang Lu, Hongyu Xu, Yiwei You, Qingzhi Wang

**Affiliations:** 1 Department of Pathology, Fuwai Hospital, Peking UnionMedical College, Chinese Academy of Medical Science, Beilishi Road No. 167, Xicheng District, Beijing, 100037 China; 2 Center for Adult Surgery, Fuwai Hospital, Peking UnionMedical College, Chinese Academy of Medical Science, Beilishi Road No. 167, Xicheng District, Beijing, 100037 China

**Keywords:** Porcine aortic valve, Structural valve deterioration, Histopathology, Macrophages, Endothelialization

## Abstract

**Background and objective:**

Bioprostheses are the most common prostheses used for valve replacement in the Western medicine. The major flaw of bioprostheses is the occurrence of structural valve deterioration (SVD). This study aimed to assess the pathological features of porcine aortic valve (PAV)-SVD based on histomorphological and immunopathological characteristics of a large cohort of patients.

**Methods:**

Histopathological data of 109 cases with resected PAV were collected. The type and amount of infiltrated cells were evaluated in the different types of bioprosthetic SVD by immunohistochemical staining.

**Results:**

The most common cause of SVD was calcification, leaflet tear, and dehiscence (23.9%, 19.3%, and 18.3%, respectively). Immunohistochemical staining demonstrated that macrophages were infiltrated in the calcified, lacerated and dehiscence PAV, in which both M1 and M2 macrophages were existed in the calcified PAV. Importantly, the higher content of M1 macrophages and less content of M2 macrophages were found in the lacerated and dehiscence PAV, and MMP-1 expression was mainly found in the lacerated PAV. The endothelialization rate of leaflet dehiscence was higher than that of calcified and lacerated leaflets. A large number of CD31+/CD11b+ cells was aggregated in the spongy layer in the lacerated and dehiscence PAV.

**Conclusion:**

Cell regeneration and infiltration is a double edged sword for the PAV deterioration. Macrophage infiltration is involved in the different types of SVD, while only MMP-1 expression is involved in lacerated leaflets. The macrophage subtype of circulating angiogenic cells in dehiscence and tear PAV could be identified, which could reserve macrophages in the PAV-SVD.

## Introduction

Valve replacement is a common surgical procedure in the treatment of valvular diseases, and about 200,000 cases have been reported worldwide. Especially in the last decade, under the influence of the aging of the population, associated with the continuous improvement of the hemodynamic performance of commercial biological prostheses [1], biological prostheses are more favored compared with mechanical prostheses. After the long-term application, bioprosthetic valves caused structural deteriorations, including calcification, narrowing of heart valve, and tearing of leaflets [2]. The crosslinking with glutaraldehyde has noticeably attracted clinicians’ attention [3], and various methods have been developed to ensure that the calcification can be effectively avoided [4]. However, the processes of tearing and dehiscence of leaflets have been neglected. The porcine aortic valve (PAV) tear or dehiscence was more prevalently reported than bovine pericardial valve (BPV) replacement [5]. The structural valve deterioration (SVD) is typically diagnosed using echocardiography and surgical observations, leading to incomplete understanding the mechanism of SVD. The present study aimed to analyze the pathological features of PAV-SVD, especially leaflet tearing and dehiscence, based on histomorphology and immunopathology of a large cohort of patients.

Bioprosthetic heart valves are fabricated from bovine/porcine pericardium or porcine heart valves and undergo SVD over time due to the lack of a regenerative capability upon chemical fixation [6, 7]. However, it has been recently reported that native valve calcification and myxomatous degeneration are active processes. The inflammatory cells, valvular interstitial cells (VICs), and valvular endothelial cells (VECs) are all involved in an active process [8–10]. Hence, the present research concentrated on the study of the role of active biological processes in PAV-SVD and investigation of the preventive measures for leaflet tearing and dehiscence.

## Methods

### Patients

Data of patients who underwent PAV replacement in our institution between January 1, 2006 and December 31, 2020 were retrospectively analyzed. The present study was approved by the Ethics Committee of Fuwai Hospital (Beijing, China). Informed consent was obtained from all patients, and the study was conducted in accordance with the principles of the Declaration of Helsinki. The normal control bioprosthesis at "time 0" of implantation could not be obtained. Participants with pannus overgrowth and paravalvular leakage were assigned into control group.

### Pathological observations

All PAV tissues were fixed with formalin, dehydrated, and wrapped with paraffin. Slides with thickness of 4–5 μm were sectioned, deparaffinized, and stained with hematoxylin and eosin (H&E) and elastic Verhoeff-Van Gieson. The histological features were observed by Zeiss microscopy, including the disruption of collagen and elastic fibers, collagen delamination, plasma protein permeation, etc. The modes of bioprosthetic valve failure were recorded according to the following recommendation [6]: (1) Structural valve dysfunction: intrinsic permanent valve alterations that could lead to hemodynamic or clinical dysfunction, including calcification, leaflet tearing, dehiscence, wear, fracture, poppet escape, and embolization. (2) Nonstructural dysfunction can lead to changes in hemodynamics, such as pannus, suture entrapment, paravalvular leakage, and proportional imbalance that are non-intrinsic. (3) Valve thrombosis: any thrombus not caused by infection attached to or near an operated valve that occludes part of the blood flow path, interferes with valve function, or is sufficiently large to warrant treatment. (4) Endocarditis: an infection of the inner lining of the heart chambers and valves.

### Quantification of collagens

The density of types I and III collagen in valve tissue slices was measured using Picrosirius red staining, followed by observation using polarized light microscopy as previously reported [11]. Briefly, type I collagen showed a red-yellow double refraction and type III collagen illustrated a red-green double refraction by Picrosirius red staining, and observed by polarized light microscopy. We measured the areas of types I and III collagen by the digital planar measurement, and their ratio was calculated by dividing the calculated area of type I collagen by the area of type III collagen. The measurement of areas of types I and III collagen was carried out using Image-Pro Plus Image Analysis software (Ver. 6.0; Media Cybernetics, Inc., Waltham, MA, USA). The measurement was conducted as follows: (1) Open the file of image of Picrosirius red staining specimen, find the count/size option, and select the yellow or green color with straw. (2) Close this window, press the "Count" button to calculate the area of the selected color. (3) Open the "view" option and find the "Statistics", then, the area of selected color is shown in the "Sum".

### Immunohistochemistry (IHC)

IHC was performed in calcified, dehiscence, tear and non-SVD-PAVs with inclusion of 10 cases in each group. The valve tissue section (5 μm) was incubated with specific primary antibodies, including rabbit smooth muscle actin monoclonal antibody (1:200), rabbit vimentin monoclonal antibody (1:200), rabbit anti-CD68 monoclonal antibody (1:8000), rabbit anti-CD16 monoclonal antibody (1:100), rabbit anti-CD14 monoclonal antibody (1:100), rabbit anti-CD163 monoclonal antibody (1:500), rabbit anti-CD11b monoclonal antibody (1:1000), rabbit anti-MMP-1 monoclonal antibody (1:100), rabbit anti-CD34 polyclonal antibody (1:150), rabbit anti-CD34 polyclonal antibody (1:150), rabbit PCNA polyclonal antibody (1:200), rabbit Ki-67 polyclonal antibody (1:200), and rabbit ICAM-1 polyclonal antibody (1:50). Except for rabbit ICAM-1 polyclonal antibody that was purchased from Proteintech Group, Inc. (Rosemont, IL, USA), other antibodies were purchased from Cell Signaling Technology, Inc. (Danvers, MA, USA). Then, the tissue sections were incubated with the IHC Detection Reagent (HRP, Rabbit; Cell Signaling Technology, Inc.) according to the manufacturer’s instructions. Slides were then developed with 3,3-diaminobenzidine (DAB) kit (Thermo Fisher Scientific Inc., Waltham, MA, USA), and counterstained with weak hematoxylin. In accordance with Taghavi-Moghadam et al.’s method, the following markers were used to identify macrophage subtypes: CD68^+^ and CD163^−^ for identification of M1 macrophages; CD68^+^ and CD163^+^ for identification of M2 macrophages [12]. The number and extent of inflammatory cell infiltrates in the bioprosthetic vlave were semi-quantified [13]. Briefly, score of 0 point indicates the absence of cells, score of 1 point indicates the existence of < 25 cells/high-power field (HPF), score of 2 points indicates the existence of 25–99 cells/HPF, score of 3 points indicates the existence of 100–249 cells/HPF, and score of 4 points indicates the existence of ≥ 250 cells/HPF. The endothelialization rate was calculated by dividing the endothelium area by the total area of leaflet surface.

### Statistical analysis

The data were expressed as the mean ± standard error of the mean (SEM). The two-tailed Student’s t-test and Levene’s test and Mann–Whitney U test were used to compare data with the Gaussian distribution and the non-Gaussian distribution. One-way analysis of variance (ANOVA) was employed for making multiple comparisons. Percentages of different types of SVD in mitral and aortic bioprostheses were compared using cross-stabbing. The variables included ratio of type I collagen to type III collagen and the immune cell infiltration grade.

## Results

### Patients’ clinical features

In Fuwai Hospital, within 3000 cases per year underwent valve replacement, 17.7% of prostheses were bioprosthetic valves. A total 143 cases of bioprostheses were enrolled, including 109 cases of PAV and 34 cases of BPV. Besides, 109 cases of PAV were involved in the study with the average age of 56.77 ± 17.40 (range 5–78) years old, of whom 36.7% were male and 63.3% were female. The average lifespan was 8.74 ± 3.75 (range 0.4–18) years. There was no significant difference in the lifespan between the mitral bioprosthetic valves and the aortic bioprosthestic valves (8.78 ± 3.75 vs. 8.69 ± 4.48 years, *P *= 0.909).

### Mode of PAV failure

The 109 PAVs included 65 mitral valves, 40 aortic valves, and 4 tricuspids. The most common cause of PAV failure was calcification (23.9%, 29/109), followed by leaflet dehiscence (19.3%, 21/109), tear (18.3%, 20/109), infective endocarditis (10.1%, 10/109), pannus (9.2%,12/109), calcification accompanied by leaflet tear or dehiscence (4.6%, 4/109), thrombus (4.6%, 4/109), accompanied surgery (6.4%, 7/109), and paravalvular leak (0.9%, 1/109). The percentage of mitral PAV leaflet dehiscence was higher than aortic PAV (30.8% vs 5.0%, χ^2 ^= 9.928, *P *= 0.001). There was no statistically significant difference in the rates of tearing or calcification of mitral and aortic valves (20.0% vs. 17.5%, χ^2 ^= 0.100, *P *= 0.751; 33.8% vs. 20.0%, χ^2 ^= 2.326, *P *= 0.127).

### Histological findings of PAV with calcification, tear and dehiscence

**Fig. 1:**
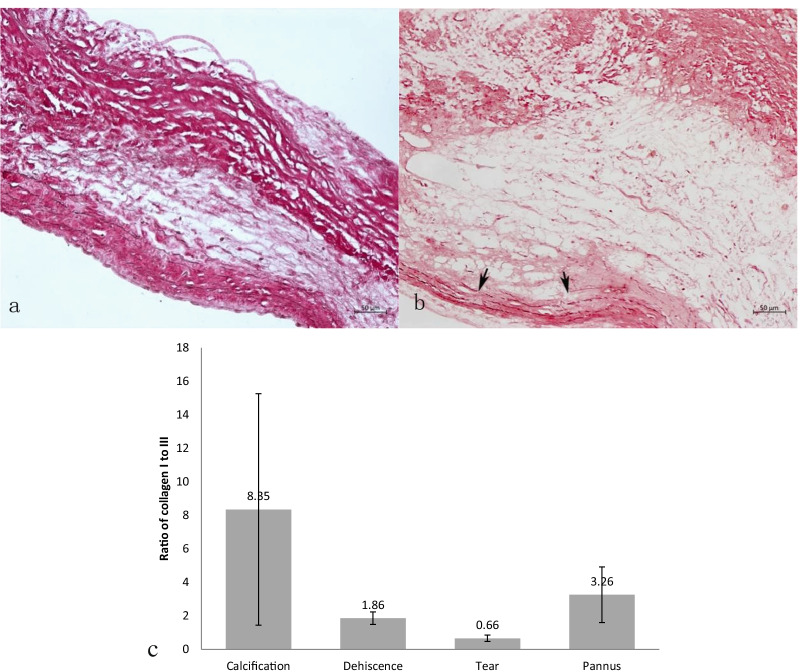
The density of collagen and elastic fibers and their arrangement in different modes of PAV failure. **a** Normal PAV showed intact leaflet structure with type I collagen located in dense layer and ventricular layer. Elastic fibers were located in ventricular layer in normal PAV (elastic Verhoeff-Van Gieson staining; original magnification, 200×). **b** Elastic fibers were broken into debris in lacerated leaflets (arrow showed, elastic Verhoeff-Van Gieson staining; original magnification, 200×). **c** The ratio of type I collagen to type III collagen was the lowest in the lacerated PAV compared with the calcified PAV (*P < 0.05, compared with calcification)

Leaflets are presented in an intact structure in non-SVD-PAVs (pannus and paravalvular leak). Type I collagen, elastic fibers, and type III collagen are located in dense layer, ventricular layer, and sponge layer, respectively, which is similar to PAV in the normal state (Fig. 1a). The lacerated leaflets exhibited collagen delamination and elastic fibers also showed breaking or disappeared (Fig. 1b). Plasma protein permeating was observed. Using Picrosirius red staining and polarized light microscopy, type I collagen I in dense layer and ventricular layer was delaminated and fractured into debris, and more amount of type III collagen was found in all three layers of the lacerated leaflets. Collagen delamination generally occurs in the hinge region of the dehiscence leaflets. The increase of type III collagen was also observed, although the degree was milder than that in the lacerated PAV. In the most of calcified PAVs, the leaflets showed the collagen deposition in dense layer. Compared with PAV with leaflet tear and dehiscence, the amount of type I collagen increased around the calcified area in PAV, and the ratio of type I collagen to type III collagen was elevated (8.35 ± 6.91 vs. 1.09 ± 0.37 and 0.66 ± 0.19, *P *= 0.016 and 0.044, respectively) (Fig. 1c). There was no significant difference in ratio of type I collagen to type III collagen between PAV with leaflet tear and dehiscence (1.09 ± 0.37 vs. 0.66 ± 0.19, *P *= 0.902).

### Infiltration and endotheliaziation of macrophages in PAV with calcification, tear and dehiscence

**Fig. 2:**
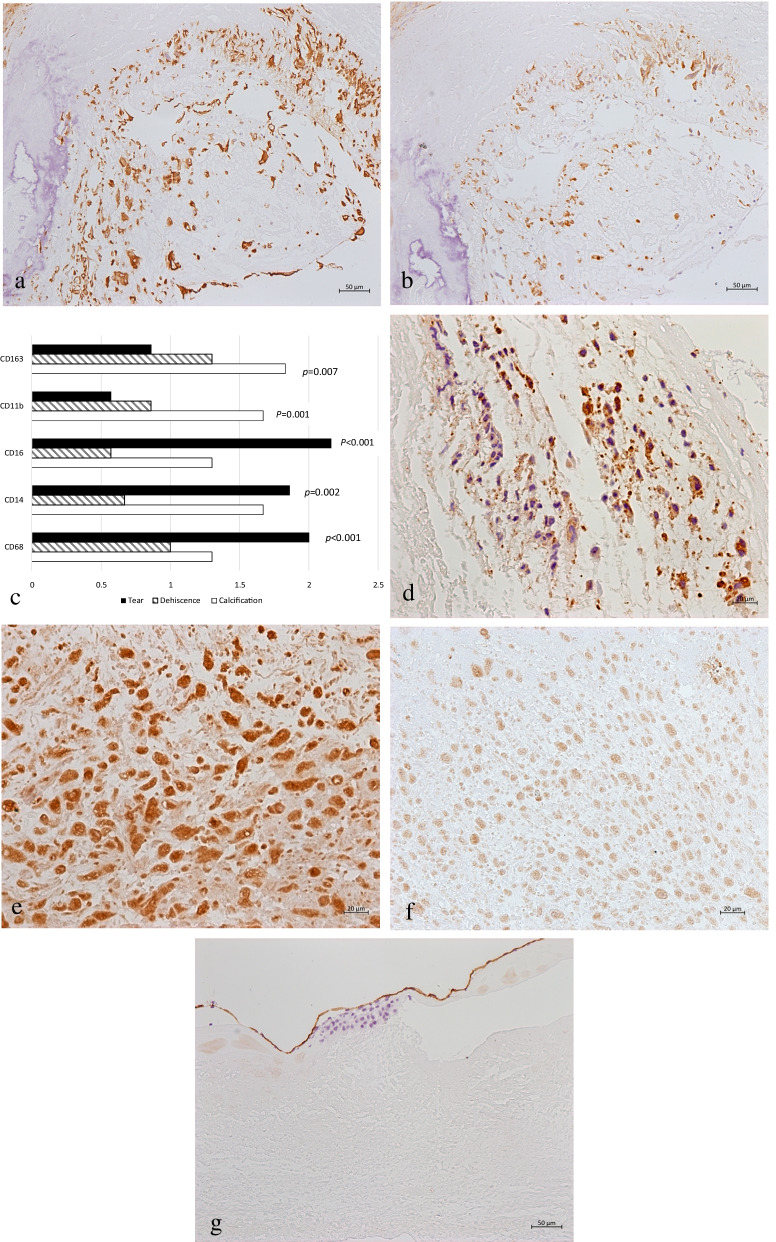
Macrophage infiltration and endothelialization in different types of PAVs. **a** VICs expressed vimentin and were aggregated around the calcified area in the PAV (original magnification, 200); **b** CD68 positive macrophages infiltrated in the damage area of PAV. **c** The grade of macrophage subtypes in calcification, dehiscence and tear PAV. **d** Macrophages expressed MMP-1 (original magnification, 200); **e** CD31 positive cells were aggregated in the spongy layer of the lacerated leaflets (original magnification, 400); **f** CD11b positive cells were aggregated in the spongy layer of the lacerated leaflets (original magnification, 400); **g** In control PAV, endothelial cells were located on the surface of cusp with CD34-positive expression (original magnification, 200)

IHC revealed an aggregation of vimentin (VIM)-positive cells in calcified, lacerated and dehiscence PAV. Most of VIM-positive cells also showed CD68 positive, which indicated that macrophages were existed objectively. However, these cells did not express smooth muscle actin (SMA) (Fig. 2a, b). Monolayer CD31(+) cells mainly attached to the surface of leaflets, which showed the morphology of endothelial cells with the positive expression of ICAM-1. The endothelialization rate of dehiscence leaflets was higher than that of calcified and lacerated leaflets (54.00 ± 27.01% vs. 25.20 ± 23.24% and 23.91 ± 25.82%, *P *= 0.033 and 0.051, respectively). PAV exhibited a significant macrophage infiltration in the calcified, lacerated and dehiscence leaflets, especially in the delaminated or calcified area or the surface of the leaflets. The grade of total macrophage infiltration, which was indicated by CD68+, was significantly higher in lacerated leaflets than that in dehiscence and calcified leaflets (2.00 ± 0.00 vs. 1.00 ± 0.00 and 1.3 ± 0.6, *P *< 0.001). Macrophages with CD14+ and CD16+ were mainly found in the lacerated leaflets compared with the dehiscence leaflets (1.86 ± 0.69 vs. 0.67 ± 0.52 and 2.14 ± 0.38 vs. 0.67 ± 0.52, *P *= 0.002 and < 0.001, respectively). Macrophages with CD11b+ and CD163+ were mainly identified in the calcified leaflets compared with the lacerated leaflets (1.67 ± 0.52 vs. 0.57 ± 0.53 and 1.83 ± 0.41 vs. 0.86 ± 0.69, *P * = 0.001 and 0.007, respectively) (Fig. 2c). MMP-1 was expressed in lacerated leaflets, and the expression level was higher than that in dehiscence leaflets (1.11 ± 0.98 vs. 0.17 ± 0.40, *P* = 0.037) (Fig. 2d). Some macrophages expressed PCNA, rather than Ki-67.

A large number of CD31 positive cells were aggregated in the spongy layer of the lacerated and dehiscence PAV, rather than in calcified and non-SVD-PAV (Fig. 2e) with the cell morphology of round or oval shape. In some of these cells, CD11b was also weakly expressed (Fig. 2f), rather than other markers of macrophages, including CD68, CD163, CD14, and CD16. The markers of endothelial cells, including ICAM-1 and CD34 were also negatively expressed. The grade of infiltrated CD31+/CD11b+ cells was higher in the lacerated and dehiscence leaflets compared with that in the calcified leaflets (2.17 ± 1.19 and 2.60 ± 0.89 vs. 0.83 ± 0.94, *P *= 0.006 and 0.003 respectively). In the control group, the PAV with pannus and paravalvular leakage demonstrated that few macrophages were located in the spongy layer, which indicated that all macrophage markers and endothelial cells were located on the surface of leaflets with the positive staining of CD31, CD34, and ICAM-1 (Fig. 2g).

## Discussion

It is considered that the bioprosthetic valve dehiscence is due to mechanical stress. It is noteworthy that the incidence of dehiscence is significantly higher in the mitral valve than that in the aortic valve, which was demonstrated in the present study. When the left ventricle contracts, the mitral valve closes and the aortic valve opens. At this moment, the left ventricle contracts to pump the blood to the aorta. The high level of left ventricular systolic pressure acts on the ventricle side of hinge region of mitral bioprosthesis. Repeated action on this region accelerates the disruption glutaraldehyde-fixed cusps, especially near the commissure, resulting in dehiscence [14]. However, there has been a paucity of publications to indicate the changes of valve histological structure with dehiscence in PAV. Mao et al. found that the microstructural damages occurred in pericardial patch during manufacturing process may identify the vulnerable sites that play an important role in the cusp dehiscence of bovine pericardial valves [15]. As an important part of pericardial patch, manufacturing process may cause the elimination of collagen bundle ripple or layered damage around the scaffold [15]. The above-mentioned research showed that there may be corresponding changes at the suture around the stent. The key factors causing the relative concentration of stress mainly include uneven or mismatched stent, suture hole, and joint [15]. In the present study, we found that the collagen delamination occurred in the hinge region of the dehiscence PAV leaflets. The increase of the amount of type III collagen was also observed, although the degree was milder than in the lacerated PAV. The extracellular matrix (ECM) damage on the suture around the stent was caused by similar biomechanical features.

Differing from dehiscence, lacerated leaflets showed the overall structural damage in valve, including collagen disruption into debris and plasma protein permeation as demonstrated in the present study. Increased content of type III collagen and decreased ratio of collagen I/III were also found in the lacerated leaflets. The collagen matrix of normal aortic valves was mainly formed by type I collagen (70%), and 25% of type III collagen [16]. Purushothaman et al. [17] showed that increasing the density of type III collagen and proteoglycan on the basis of reducing the density of type I collagen may cause the expansion of sponge layer and affect the function of mitral valve. It was found that low-density type I collagen would damage the formation of thick functional type I collagen fibers. However, type III collagen has a better ductility than thick type I collagen. The intuitive response to injury is manifested in the synthesis of type III collagen [18].

At present, the clinically available PAV is mainly cross-linked by glutaraldehyde (GLUT) to improve the ECM stability and reduce the immunogenicity of valve tissue. However, the residual GLUT is cytotoxic, which can reduce the adhesion and proliferation of endothelial cells, and hinder the endothelialization of valves [19, 20]. Additionally, the residual GLUT in PAV can also activate macrophages, phagocytes, T lymphocytes, and aggregation of platelet [21–23]. However, few studies have investigated the distribution of subtypes of macrophages in SVD-PAV. In the present study, we found that all subtypes of macrophages, including M1 and M2, were observed surrounding and apart from the calcification area. In lacerated PAV, macrophages with CD14+ and CD16+ were mainly found in the lacerated leaflets compared with the dehiscence leaflets. Macrophages with CD11b+ and CD163+ were mainly identified in the calcified leaflets compared with the lacerated leaflets. Macrophages are typically classified into M1 and M2 subtypes [24]. Some scholars demonstrated that M1 macrophages cause inflammation and M2 macrophages suppress differentiation and activation of M1 macrophages [25, 26]. Several studies have concentrated on the myxomatous mitral valve degeneration (MVD), which is characterized by leaflet thickening, diffuse accumulation of proteoglycan, collagen fiber disruption, and elastin fragmentation. These histological features are similar to the lacerated PAV as observed in the present study. Increased macrophage numbers in association with valve myxomatous ECM changes are common to MVD arising from various causes [27, 28]. Kim et al. found that mitral valve obtained from gene-edited Marfan's syndrome (MFS) pigs and human MFS patients exhibited increased monocytes and macrophages with expressions of CD14, CD64, CD68, and CD163 [29]. Additionally, comparative transcriptomic evaluation of both genetic and acquired forms of MVD revealed a remarkable upregulated inflammatory response in diseased valves. They also found that deficiency of monocytes was protective against MVD progression, resulting in a minimal leaflet thickening and preserved mitral valve integrity [29]. CD163 is the marker of M2 macrophages, which are capable of anti-inflammatory responses and repair damaged tissues [30, 31]. CD11b, which is the α-chain of intergrin receptor, is mainly expressed in the surface of innate immune cells, including macrophages and neutrophils. CD11b is crucial for maintaining homeostasis and tolerance, as well as downregulating inflammatory mechanisms. Macrophages deficient in CD11b have enhanced activation of nuclear factor-κB (NF-κB) and other Toll-like receptor (TLR)-dependent pathways and production of inflammatory cytokines [32, 33]. Combined with previous studies, we conclude that the imbalance infiltration between M1 and M2 macrophages in the leaflets is the essential mechanism of PAV laceration. Another important finding is that PCNA expression was found in some macrophages, while Ki-67 was negatively expressed in these cells. The Ki-67 index is a marker used to evaluate cell proliferation activity. An increase in Ki-67 expression indicates a rise of the mitotic activity and the cell proliferation. While the half-life of PCNA is approximately 20 h, and staining has been found in cells during the S phase. Therefore, PCNA was used as a marker to identify cells that were actively undergoing or have recently undergone proliferation [34, 35]. In the present study, macrophages with PCNA+ and Ki67- may indicate that they have recently undergone proliferation, rather than mitosis. On the other hand, the positive expression of PCNA indicates that the macrophages are recruited macrophages since the resident cells in glutaraldhyde-treated cusps will not be proliferating.

Proteolytic enzymes, such as matrix metalloproteinases (MMP-1, MMP-2, MMP-9, and MMP13) and cathepsin K, have also been detected during diseases associated with pathologic remodeling [35, 36]. The local balance between MMPs and tissue inhibitors of metalloproteinases (TIMPs) plays a decisive role in tissue remodeling [37, 38]. Among them, MMP-1 arises from macrophages and fibroblasts. In terms of affinity, collagen fibers are the most obvious. It is conducive to activate type I collagen decomposition [37, 38]. Few studies have been performed to investigate the role of MMP-1 in the SVD PAV. Our study showed that MMP-1 was mainly expressed by macrophages in the lacerated PAV and the damaged area in the dehiscence PAV. Previous studies have mainly concentrated on the MMP-1 and native valve calcification, and showed a correlation between MMP-1 level and heart valve stenosis [16, 39]. Inconsistently, we did not find the increased MMP-1 expression in calcified PAV, although the macrophage infiltration was obvious. This discrepancy demonstrates that the mechanism of calcification of bioprostheses does not depend on the MMP-1 expression. Other family members of MMPs should be investigated in the future. How to decrease the suture area in the bioprostheses and anti-inflammatory therapy are effective methods to alleviate the dehiscence PAV.

In the present study, we also found that the macrophages were involved in the calcification of bioprostheses. M1 and M2 macrophages were both found in the calcified area of leaflets. Compared with lacerated leaflets, calcified leaflets had more infiltrated M2 macrophages, which could indicate that the balance between proinflammation (M1) and antiinflammation (M2) controls the remodeling and calcification of the valve. It can also be concluded that bioprosthese calcification is the repair reaction after ECM degeneration mediated by macrophages. One of the important findings of the present study was the existence of VIM(+)/SMA(-) cells, and the latter is one of the biomarkers of VICs in native heart valve. It was reported that VICs derived from human pluripotent stem cells (hPSCs) could be controlled by several pathways, including BMP, FGF, Wnt, etc. [40–42]. Jiao et al. found that hPSCs with truncated Notch1 have impaired smooth muscle cell and endothelial differentiation, which indicated the decreased expression levels of SMA and CD31 [43]. Relevant studies have shown that the formation of thoracic aortic aneurysm in some patients could be caused by Notch1 gene mutation affecting the SMC differentiation of bicuspid aortic valve [43]. There has been a paucity of studies concentrated on the mechanism and role of immature VICs in PAV. Thus, further investigation on the pathways of the derivation of VICs needs to be performed. However, these immature VICs still have their essential functions, including inducing calcification of bioprostheses by VICs, which could be pathologically differentiated into myofibroblast-like VICs or osteoblast-like VICs.

It was found that a large number of CD31+ and CD11b+ cells existed in the sponge layer of PAV. CD31 is the surface marker of circulating angiogenic cell (CAC) [40–43]. The major subtypes of CD31+ CACs are T-cells (CD3+) [16, 40–43], monocyte/macrophages (CD14+ and CD11b+) [42, 43], and progenitor cells (CD34+) [44–46]. To date, few studies have concentrated on the role of CD31+/CD11b+ cells in the valvular heart diseases. Kim et al. found that surface markers co-expressed on CD31+ cells from coronary artery disease patients were downregulated in T-cell populations (CD3+) and upregulated in inflammatory cell populations (CD14+ and CD11b+) compared with in healthy controls. They demonstrated that the number of CD31+ cells might predict adverse cardiovascular events [16]. Oba et al. found that in the non-calcified aortic valve, the levels of markers of macrophage subtypes, including CD68, CD163, and CD206 were elevated in the spongiosa, which may suggest that the sponge layer may provide a microvascular network for infiltration of macrophages [47]. Compared with the calcified PAV, the lacerated and dehiscence leaflets had wider spongiosa, which accommodate more CD31+/CD11b+ circulating angiogenic cells. The problem is that whether these CD31+/CD11b cells could be the predictor of bioprosthesis inflammation. The exact role of these cells in PVA has not yet been clarified, and further research is needed. One possible role of these cells is the reserve of macrophages with pro-immflammatory or anti-inflammatory functions in the PAV-SVD.

Endothelia cells play a key role in reducing calcification and degeneration by acting as an effective barrier between the valve and the blood to prevent plasma from penetrating into the valve tissue [48–50]. Additionally, the growth and reproduction of endothelial cells on PAV are also conducive to tissue regrowth and repair. Aldehydes remaining in GLUT-treated PAV are significantly toxic to cells and mainly have a poor endothelialization ability. Xu et al. found that PAV treated by radical polymerization crosslinking exhibited better cytocompatibility and endothelialization potential *in vitro* [51]. In the present study, we found a relatively higher endothelialization rate in the dehiscence leaflets than that in the calcified and lacerated leaflets, which indicated the protective role of endothelia against the calcified leaflets and lacerated lesion. Endothelialization is another factor influencing the SVD, especially leaflet tear and calcification. Our study suggested that improving the endothelialization by different methods may be advantageous to prevent the bioprosthesis from leaflet tear and calcification.

There were certain limitations in the present study. First, we did not analyze the characteristics of different brands of commercial bioprosthetic valves because of the limited sample size. There must be dissimilarity in the histological features of different brands. It is therefore necessary to collect more bioprosthetic valves to analyze respectively. In addition, in the present study, we found CD31+/CD11b+ cells in the tear and dehiscence PAV, and deduced that these cells were macrophage subtype of circulating angiogenic cell (CAC), which is consistent with previous studies. Additional research should be performed to identify these cells and to reveal the functions of these cells in the pathogenesis of SVD in the future.

## Conclusions

In summary, through the investigation of a series of SVD cases, the histopathological characteristics of PAV were deeply analyzed. It was revealed that the changes of collagen type and density and infiltration of macrophages accompanied by MMP-1 expression are the major histological markers, which can be valuable to clarify the pathogenesis of SVD. The different subtypes of macrophages existing in the calcified and tear or dehiscence PAV demonstrated the imbalance infiltration between M1 and M2 macrophages in the leaflets, which is the essential mechanism of PAV laceration. The macrophage subtype of circulating angiogenic cells in dehiscence and tear PAV could be identified, which could reserve macrophages in the PAV-SVD.

## Data Availability

All data generated or analysed during this study are included in this published article. My data do not include "DNA and RNA sequences, Genomics and transcriptomics datasets,Linked phenotype and genotype data for human subjects and Gene expression data".

## Abbreviations

SVD: Structural valve deterioration
PAV: Porcine aortic valve
BPV: Bovine pericardial valve
VICs: Valvular interstitial cells
VECs: Valvular endothelial cells
GLUT: Glutaraldehyde
MVD: Mitral myxomatous degeneration
MFS: Marfan’s syndrome
hPSCs: Human pluripotent stem cells
CAC: Circulating angiogenic cell
